# Real‐Life Functioning in 22q11.2 Deletion Syndrome in Relation to Neurocognitive Abilities and Psychotic Symptoms: A Comparison With Idiopathic Schizophrenia

**DOI:** 10.1111/jir.13200

**Published:** 2024-11-29

**Authors:** Tommaso Accinni, Marianna Frascarelli, Pierluigi Cordellieri, Georgios D. Kotzalidis, Martina Fanella, Carlo Di Bonaventura, Carolina Putotto, Bruno Marino, Paola Bucci, Luigi Giuliani, Annalisa Maraone, Massimo Pasquini, Fabio Di Fabio, Antonino Buzzanca, the Italian Network for Research on Psychoses

**Affiliations:** ^1^ Department of Human Neurosciences, Faculty of Medicine and Dentistry Sapienza University of Rome Rome Italy; ^2^ Department of Psychology Sapienza University of Rome Rome Italy; ^3^ Department of Neurosciences, Mental Health, and Sensory Organs, Faculty of Medicine and Psychology Sapienza University of Rome Rome Italy; ^4^ Department of Pediatrics, Obstetrics, and Gynecology Sapienza University of Rome Rome Italy; ^5^ Department of Psychiatry University of Campania “Luigi Vanvitelli” Naples Italy

**Keywords:** 22q11.2 deletion syndrome, neurocognition, psychosis, real‐life functioning, schizophrenia, social functioning

## Abstract

**Background:**

The 22q11.2 deletion syndrome (22q11.2DS) entails intellectual disabilities and higher risk of psychotic disorders. Neurocognitive deficits predict real‐life functioning of schizophrenic patients. We investigated real‐life functioning in 22q11.2DS, aiming at defining how neurocognitive profile and psychopathological variables impact on psychotic patients' social functioning.

**Methods:**

We recruited 63 patients with schizophrenia (SCZ, *N* = 63), 44 with 22q11.2DS (DEL, *N* = 44) and 19 with 22q11.2DS and psychosis (DEL–SCZ, *N* = 19), all matched for age, sex and neurocognitive profile; we administered the Positive and Negative Syndrome Scale (PANSS), the Brief Negative Symptom Scale (BNSS), the Specific Levels of Functioning (SLoF) scale and the Measurement and Treatment Research to Improve Cognition in Schizophrenia Consensus Cognitive Battery (MCCB). We implemented descriptive analyses, MANCOVA and linear regression statistics.

**Results:**

The DEL–SCZ and the SCZ groups showed similar levels in *Interpersonal Relationships* (*p* = 0.093) and *Social Acceptability* subscales (*p* = 0.283). The DEL group scored higher on the *Interpersonal Relationships* subscale compared with the SCZ group (*p* = 0.001). The groups scored similarly on the other SLoF subscales. Both BNSS total score (beta = −0.343; *p* = 0.004) and BNSS asociality (beta = −0.487; *p* = 0.038) significantly predicted the *Interpersonal Relationships* variable in the groups with psychosis (SCZ and DEL–SCZ).

**Discussion and Conclusions:**

Individuals with 22q11.2DS display a similar real‐life functioning to patients with chronic schizophrenia. *Social functioning* impairments are typical of psychosis regardless of the genetic condition and highly predicted by negative symptoms like asociality. The 22q11.2DS represents a reliable biological model to study vulnerability to psychosis and its consequences on patients' real‐life and social functioning.

## 1 Background

The autosomal dominant microdeletion at the 11.2 strand on the long arm (q) of chromosome 22 is the most common among known rare copy number variations (CNVs) (McDonald‐McGinn et al. 2015) (Olsen et al. 2018); it causes the 22q11.2 deletion syndrome (22q11.2DS), which is a multisystem syndrome with an incidence ranging from 1:3000 to 1:6000 of new births (Olsen et al. 2018). The 22q11.2 microdeletion is hemizygotic, involving about 40 coding genes with a large phenotypic expression (Zamariolli et al. 2023). 22q11.2DS includes different clinical conditions deriving from a common neurodevelopmental defect that affects the neural crest (Ardinger and Ardinger 2002). Congenital heart defects, thymus hypoplasia with primary immunodeficiency and palatal defects have been reported (Bamforth and Burn 2013). Interestingly, intellectual disabilities characterize the clinical phenotype of 22q.2DS which additionally has been tightly related to neuropsychiatric condition with particular regard to schizophrenia. In fact, individuals with 22q11.2DS show an increased risk of developing a psychotic illness during their lifespan, with rates ranging in various studies from 23% to 43% (Fiksinski et al. 2023; Murphy, Jones and Owen 1999; Provenzani et al. 2022; Schneider et al. 2014). Psychotic symptoms in 22q11.2DS have been described as comparable with those of schizophrenia without a clear genetic aetiology; thus, this syndrome may represent a reliable model for studying the neurobiological underpinnings of schizophrenia and psychotic disorders, in general (Bassett et al. 2003).

People with schizophrenia suffer from a significant impairment of their independent living, without achieving an expected level of autonomy concerning productive activities and social abilities (Galderisi et al. 2014). For these reasons, functional outcomes and functional recovery in patients with schizophrenia are increasingly addressed as main treatment targets. Therapeutic programmes have classically involved symptom management as the positive ones are related to thought disorder and abnormal perceptions and the negative ones concern symptoms like blunted affect, apathy, alogia, anhedonia or asociality (American Psychiatric Association 2013). Currently, they seem to increasingly focus on patients regaining social and occupational functioning (Lahera et al. 2018). Rehabilitation programmes such as social skills training and cognitive remediation, are increasingly adopted in the frame of modern therapeutic processes enabling persons with psychosis to achieve a sufficient level of functioning (Greenwood, Landau and Wykes 2005; Kopelowicz, Liberman and Zarate 2006).

Evidence is accumulating on the likelihood of a mutual influence between neurocognition, psychopathology and real‐life functioning. In fact, neurocognitive deficits, negative symptoms and depression have been associated with poor functioning levels in patients with psychosis displaying significantly impaired independent life (Bowie and Harvey 2005). More precisely, social cognition and negative symptoms resulted to longitudinally impact patients' interpersonal functioning, whereas nonsocial cognition would rather influence everyday life skills; both social and nonsocial cognition affects professional skills (Giuliani et al. 2021). Interestingly, negative more than positive symptoms are strongly related to longitudinal functioning outcomes (Herbener and Harrow 2004), being associated with poorer insight and impaired coping strategies and leading to worse quality of life (Montemagni et al. 2014). Of note, neurocognitive deficits appear to be a better predictor of functional outcomes in patients with schizophrenia than other illness dimensions (Bowie and Harvey 2006). Moreover, impaired premorbid academic and social functioning in patients with schizophrenia may represent a vulnerability marker, which is further associated with negative symptom severity and neurocognitive impairment (Bucci et al. 2018).

Cognitive profiles have been thoroughly described for 22q11.2DS, showing a significant lifespan variability both inter‐individually and intra‐individually (De Smedt et al. 2007; Jacobson et al. 2010). Starting with childhood and adolescence, people with 22q11.2DS show significant cognitive impairments and intellectual disabilities (Gerdes et al. 2001; Roizen et al. 2007; Swillen et al. 1999): deficits in mathematical abilities (De Smedt et al. 2007), in visuo‐spatial memory (Vicario, Yates and Nicholls 2013), in attention (Antshel et al. 2013) and in executive functions have been described (Van Aken et al. 2010). Social cognitive deficits are particularly prominent in 22q11.2DS, even after adjusting for global intellectual function, more than in other idiopathic neuropsychiatric conditions (Jalal et al. 2021); moreover, social cognition in 22q11.2DS proved to be similarly impaired to that of people with idiopathic schizophrenia and regardless of psychotic symptoms, appearing as a potential endophenotype of psychotic vulnerability (Accinni et al. 2022). A decline in neurocognitive abilities has been strongly associated with an increased risk of psychotic onset in patients with the 22q11.2DS, preceding a full‐blown clinical presentation (Vorstman et al. 2015).

To date, not much is known about adult real‐life functioning in 22q11.2DS, with particular regard to social functioning; there is no evidence of the impact of neurocognitive impairments and symptom severity on real‐life and social functioning of people with 22q11.2DS. A widespread functional impairment has been thoroughly described, with intelligence quotient and diagnosis of schizophrenia emerging as significant predictors of patients' adaptive functioning (Butcher et al. 2012). However, further investigations are needed to deepen the role of neurocognitive and psychopathological domains in social functioning of people with 22q11.2DS.

## 2 Objectives

The first aim of the study was to investigate real‐life functioning and its subdimensional characteristics in 22q11.2DS. Patients with this syndrome may display specific levels of real‐life functioning like those of people with idiopathic schizophrenia, even before the eventual psychotic onset which in turn might worsen patients' level of functioning.

The second aim was to evaluate the impact of both neurocognitive and psychopathological variables on social functioning of the recruited sample. We hypothesized that the symptomatology would have an impact on patients' interpersonal functioning similarly to neurocognitive deficits, regardless of the underlying genetic condition.

The above evaluations could further confirm that 22q11.2DS is a reliable biological model to study genetic vulnerability to psychosis.

## 3 Methods

The sample of the study consisted of 126 individuals aged between 16 and 66 years, divided in three groups. The first consisted of 63 patients with idiopathic schizophrenia (SCZ, *N* = 63), the second of 44 patients with the 22q11.2 microdeletion (DEL, *N* = 44) and the third of 19 patients carrying the 22q11.2 microdeletion and diagnosed with a psychotic disorder (DEL–SCZ, *N* = 19, i.e., 9 patients with schizophrenia, 3 with schizophreniform disorder and 7 with a psychotic disorder not otherwise specified). These patients at the time of assessment were on stable medication in remission from the acute phase of the illness. Sample recruitment occurred at the Department of Human Neuroscience of Sapienza University of Rome[, from 2014 to 2021. Regarding the SCZ group, we selected the participants from an original sample of 921 patients with a diagnosis of schizophrenia in the Italian Network for Research on Psychoses (NIRP), a multicentre research programme arising from the collaboration of 26 Italian Universities[ (Galderisi et al. 2014). For each recruited person with the 22q11.2 microdeletion, we selected from the NIRP sample one patient with psychosis matched for sex, age and general cognitive profile, as defined by a composite score, as we will describe below. The genetic diagnosis was established through a complete genetic investigation using fluorescent in situ hybridization (FISH). Individuals with multiple genetic abnormalities were excluded. Participants with 22q11.2DS who presented a cognitive profile ranging from severe to highly severe impairment were excluded from the study cohort. Each participant signed free, informed consent. The study adopted the Principles of Human Rights, as issued by the World Medical Association at the 18th WMA General Assembly, Helsinki, Finland, June 1964 and subsequently amended by the 64th WMA General Assembly, Fortaleza, Ceará, Brazil, in October 2013. The research protocol has been reviewed and approved by the Ethics Committee of the [*omissis*], protocol. [*omissis*]. All tests and assessment of symptom severity, neurocognition and patients' real‐life functioning have been performed at the same time. All data were anonymized. For all participants, demographic data were collected, including age, sex and years of education; for clinical groups (DEL–SCZ and SCZ), data were also collected for clinical variables like age at illness onset, duration of illness and exposure to antipsychotic treatment, that is, no antipsychotics, first‐generation antipsychotics (FGAs), second‐generation antipsychotics (SGAs) or both.

### 3.1 Clinical Evaluation

Each participant underwent a clinical evaluation from a psychiatrist who assessed the psychiatric symptomatology according to the DSM‐5 criteria (American Psychiatric Association 2013). During assessment, symptom severity was assessed by the Positive and Negative Syndrome Scale (PANSS) (Kay, Fiszbein and Opler 1987) and the Brief Negative Symptom Scale (BNSS) (Kirkpatrick et al. 2011; Merlotti et al. 2014). We implemented a separate investigation of positive and negative symptoms by means of the above two psychometric tools, given they were previously shown to have different specificities in describing such symptoms (Mucci et al. 2015).

### 3.2 Neurocognitive Evaluation

The Measurement and Treatment Research to Improve Cognition in Schizophrenia Consensus Cognitive Battery (from now on MCCB) (Nuechterlein et al. 2008) was employed to evaluate patients' neurocognitive profile assessing the key cognitive domains relevant to schizophrenia. MCCB measures clinical outcomes and treatment effectiveness assessing cognitive improvement in patients with psychosis. To provide a general performance level, we used an *MCCB composite score*, consisting of the weighted average of battery subtests, except for the *Attention/Vigilance* domain, because most of the 22q11 deletion patients were not able to complete this task. The MCCB composite score derived from the remaining subtest's mean *z*‐scores were compared with the mean of the normative sample of the Italian NIRP (Galderisi et al. 2018). The MCCB composite score was aimed at adjusting the results of real‐life functioning analyses for any further potentially detectable cognitive dysfunction.

### 3.3 Evaluation of Patients' Real‐Life Functioning

The Specific Levels of Functioning scale (SLoF) was employed in our study design to assess real‐life functioning of recruited patients (Montemagni et al. 2015; Mucci et al. 2021). It is a 43‐item multidimensional behavioural interview, referring to the past week; the interview is administered to the patient's caregiver looking at three main domains, each in turn involving two specific factors that are related to the patient's functioning and independent life profile: *Self Maintenance*, including the subscales *Physical Functioning* and *Personal Care Skills*; *Social Functioning*, evaluating both *Interpersonal Relationships* and *Social Acceptability*; *Community Living Skills*, related to *Activities* and *Work Skills* subscales.

The SLoF subscales assess patients' current functioning comprising their observable behaviour without looking at psychopathology and cognitive dysfunction. The potential total scores of the scale range from 43 to 215, where the higher the Total score (SLoF Total score), the better the overall functioning of the patient.

As previous studies have shown that social functioning and social skills impairments in psychotic disorders, in the present study we focused on the SLoF *Social Functioning* section, aiming at evaluating the different groups for these specific abilities. Indeed, social functioning has been tightly associated with the psychopathological core of psychosis (Bucci et al. 2018; Dodell‐Feder, Tully and Hooker 2015); conversely, the SLoF *Self Maintenance* section may be likely influenced by the existence of significant physical inabilities that characterize people with 22q11.2DS, thus affecting the comparison between a genetic condition and idiopathic schizophrenia. Similarly, the SLoF *Community Living Skills* section evaluates abilities and performances particularly influenced by the environment and therefore less determined by pure clinical conditions.

### 3.4 Statistical Analysis

Continuous demographic and clinical variables were analysed through analysis of variance (ANOVA). Bonferroni's correction was applied to post‐hoc comparisons and when the assumption of homogeneity of variance was violated, the Games–Howell test was applied. For the variables examined only in the DEL–SCZ and SCZ groups, the *t*‐test for independent samples was used. Categorical variables were investigated through Pearson's *χ* ^*2*^ test. A multivariate analysis of covariance (MANCOVA) design was applied to test whether the scores at SLoF subscales significantly differed between the recruited groups. We considered *group* as the independent variable consisting of three levels, namely individuals with 22q11.2 microdeletion without psychosis (*DEL*), individuals with 22q11.2 microdeletion and psychosis (*DEL–SCZ*) and patients with idiopathic psychosis (*SCZ*), and 5 SLoF subscales as dependent variables, namely *Personal Care Skills*, *Interpersonal Relationships*, *Social Acceptability*, *Activities* and *Work Skills*. The SLoF physical subscale, relating to the self‐maintenance dimension, was not included in the ANOVA design to enhance interpretation as people with 22q11.2 microdeletion suffer from a multisystemic syndrome involving several biological organs and apparatuses, with significant repercussions on general physical functioning; this aspect could probably affect the SLoF subscale scores of groups with a rare genetic syndrome and patients with idiopathic schizophrenia. The MANCOVA design included *Age* and *MCCB composite score* as covariates. The analysis of effects was evaluated through Wilks' Lambda, Pilai's Track, Hotelling's Track and Roy's Root statistics; univariate effects were then examined. Planned comparisons were made to test the difference between the groups: The DEL–SCZ group was compared with SCZ and DEL groups, and the SCZ group to the DEL group. Different simultaneous linear regression models have been implemented combining groups to evaluate the impact of neurocognitive and psychopathological variables on SLoF *Self Functioning* domain, including the *Interpersonal Relationships* and *Social Acceptability* variables. We chose the *BNSS total score*, the *PANSS positive symptoms* and the *PANSS general psychopathology* scores as reliable indicators of negative, positive and general symptom severity, respectively, in patients with psychosis, as already reported (Kay, Fiszbein and Opler 1987; Kirkpatrick et al. 2011); the *MCCB composite score* represents a thorough indicator of the general cognitive level of psychotic patients, as previously reported (Accinni et al. 2022). Statistical significance was set at *p* < 0.05 for all analyses. We used the SPSS 25.0 version (Statistical Package for the Social Sciences, IBM Co., Armonk, New York, USA, 2017) for all statistical analyses.

## 4 Results

Clinical and demographic characteristics of the recruited sample are shown in Table 1.

**TABLE 1:** Demographical and clinical variables of the recruited sample (continuous variables).

Continuous variables	DEL–SCZ	SCZ	DEL	*F*	*p*
	*N* = 19	*N* = 63	*N* = 44		
Mean age ± SD	26.6 ± 7.3	24.9 ± 6.4	23.8 ± 6.6	1.256	0.289
PANSS pos^a^	18.8 ± 5.8	15.8 ± 6.2	9.9 ± 3.2	22.210	< 0.001
PANSS neg^a^	21.2 ± 5.8	23.9 ± 8.9	13.0 ± 4.7	26.881	< 0.001
PANSS pg^a^	43.9 ± 9.9	38.1 ± 12.3	30.3 ± 8.0	11.652	< 0.001
PANSStot^a^	83.8 ± 19.0	77.8 ± 23.5	53.2 ± 13.4	22.750	< 0.001
BNSS tot	36.8 ± 14.2	39.0 ± 17.8	19.5 ± 14.9	16.179	< 0.001
MCCB *comp* ^b^	44.1 ± 4.8	46.8 ± 6.0	47.2 ± 6.4	1.903	0.154
Onset	19.9 ± 5.1	20.4 ± 4.4		0.133	0.716
DoI (years)	6.6 ± 4.9	4.6 ± 4.7		2.593	0.111
Edu^a^	11.4 ± 2.5	11.9 ± 2.9	12.3 ± 1.6	1.073	0.345

Abbreviations: BNSS tot, BNSS total score; DEL, 22q11.2 deletion syndrome; DEL–SCZ, 22q11.2 deletion syndrome and schizophrenia; DoI, duration of illness; Edu, years of education; MCCB comp, MCCB composite score; ONSET, age at first psychotic episode; PANSS pos, PANSS positive symptoms; PANSS neg, PANSS negative symptoms; PANSS pg, PANSS general psychopathology; PANSS tot, PANSS total score; SCZ, schizophrenia.

^a^ Variables whose variances proved to be significantly unequal as Levene's test was significant (*post hoc* test assessed through the Games ‐Howell's test for variables with unequal variances, and with Bonferroni's correction for variables with equal variances).

^b^ *z*‐score; mean scores ± standard deviation (SD) are represented.

As expected, given the procedure of sample matching, the three clinical groups did not differ for sex, age and general cognitive profile. There were no differences in educational level across the groups. Considering the clinical variables, no statistical differences emerged concerning age at illness onset, illness duration and the exposure to drug treatments, for both first‐ and second‐generation antipsychotics.

From a psychopathological standpoint, as expected, the two groups with psychosis (SCZ and DEL–SCZ) scored significantly higher on all clinical scales compared with the DEL group; SCZ and DEL–SCZ did not differ on any psychopathological evaluation.

By means of the MANCOVA, whose assumptions were tested and met (Appendix A), a significant *group*'s effect on combined dependent variables, as indicated by Pillai's trace, was found (Tables A2 and A8). To note, the covariate *MCCB composite score* showed a significant effect on combined dependent variables, while the covariate *Age* did not (Table 2). *Social Acceptability*'s effect was not significant at univariate analyses (Table 3). These findings showed that the groups significantly differed in scores at SLoF subscales even after controlling for *Age* and *MCCB composite score*.

**TABLE 2:** MANCOVA multivariate tests with effects of fixed factors, group variables and covariates *Age* and *MCCB composite score*.

Multivariate tests	Pillai's trace	*F*	Hypothesis *df*	Error *df*	Sig.	Partial *η* ^2^
Group	0.502	7.77	10	232	< 0.001	0.251
Age	0.064	1.57	5	118	0.174	0.064
*MCCB comp*.	0.196	5.6	5	118	< 0.001	0.196

Abbreviations: *df*, degrees of freedom; MCCB comp, MCCB composite score; Sig., statistical significance.

**TABLE 3:** Scores at SLoF subscales for each group, univariate tests and Levene's tests.

SLoF Subscales	DEL–SCZ	SCZ	DEL	Univariate test *F*_2,118_	Univariate test sig.	Partial *η* ^2^	Levene's test *F*_2,123_	Levene's test sig. (*p*)
Pers. care	31.36 (11.8)	43.1 (7.4)	42.57 (9.9)	13.24	< 0.001	0.182	1.58	0.209
Interpers.	28.47 (8.8)	31.05 (9.6)	37.9 (10.0)	7.90	< 0.001	0.117	0.10	0.901
Social accept.	39.64 (11.57)	43.50 (9.2)	42.16 (10.3)	0.72	0.319	0.012	0.57	0.568
Activities	27.45 (13.4)	40.86 (6.6)	38.2 (9.3)	14.93	< 0.001	0.201	4.95	0.009*
Work skills	28.22 (11.9)	35.57 (8.6)	39.71 (9.1)	7.79	< 0.001	0.116	2.34	0.163

*Note:* Mean *T* scores and (DS) are presented. Univariate tests (*F*), sig. (*p*) and effect size (partial *η* ^2^); Levene's test (*F*) and (*p*).

Abbreviations: DEL, 22q11.2 deletion syndrome; DEL–SCZ, 22q11.2 deletion syndrome and schizophrenia; SCZ, schizophrenia; sig., significance; SLoF, Specific Level of Functioning Scale; Pers. care, SLoF Personal Care Skills; Interpers., SLoF Interpersonal Relationships; Social accept., SLoF Social Acceptability; Activities, SLoF activities; Work skills, SLoF Work Skills.

The planned comparisons implemented by the *K* matrix showed that the SCZ group performed better on the *Personal Care Skills* and *Activities* SLoF subscales, and on the *Work Skills* subscale compared with DEL–SCZ group; there were no significant differences between the DEL–SCZ and the SCZ group as regards the *Interpersonal Relationships* and *Social Acceptability* subscales. The DEL group scored higher on the *Interpersonal Relationships* and on the *Work Skills* subscales compared with the SCZ group. No significant differences between these groups emerged for other SLoF subscales (Table 4).

**TABLE 4:** Planned comparisons at the *K* matrix: Results of contrasts and univariate tests.

SLoF subscales	DEL–SCZ vs. SCZ				DEL–SCZ vs. DEL				SCZ vs. DEL			
	*F*_1,118_	Sig.	Partial *η* ^2^	*d*	*F*_1,118_	Sig.	Partial *η* ^2^	*d*	*F*_1,118_	Sig.	Partial *η* ^2^	*d*
*Pers. care*	25.52	< 0.001	0.177	−1.19	18.98	< 0.001	0.138	−1.03	0.34	0.559	0.003	0.70
*Interpers*.	0.38	0.541	0.003	−028	9.80	0.076	0.076	−1.00	12.68	< 0.001	0.096	−4.1
*Social accept*.	1.28	0.260	0.011	−0.37	0.31	0.579	0.003	−0.23	0.51	0.475	0.004	0.14
*Activities*	29.86	< 0.001	0.201	−1.27	16.31	< 0.001	0.121	−0.93	2.48	0.118	0.020	0.33
*Work skills*	6.73	0.011	0.54	−0.71	15.42	< 0.001	0.115	−1.09	4.23	0.042	0.034	−0.47

*Note:* The table shows parameters F, statistical significance, partial *η* ^2^ and Cohen's *d*, as a further measure of effect size.

Abbreviations: DEL, 22q11.2 deletion syndrome; DEL–SCZ, 22q11.2 deletion syndrome and schizophrenia; SCZ, schizophrenia; Sig., significance; SLoF, Specific Level of Functioning Scale; Pers. care, SLoF personal care skills; Interpers., SLoF interpersonal relationships; Social accept., SLoF Social Acceptability; Activities, SLoF activities; Work skills, SLoF Work Skills.

### 4.1 Social Acceptability

Concerning the *Social Acceptability* subscale, the effect of the between‐subjects test did not show significant differences between groups and the variances were equal (Levene's test not significant); therefore, the simultaneous linear regression was performed on the whole sample (DEL, DEL–SCZ and SCZ; *N* = 126); this model, employing as predictors the *BNSS total score*, the *PANSS positive symptoms*, the *PANSS general psychopathology* and the *MCCB composite score*, and the *SLoF Social Acceptability* as the dependent variable, did not result significant (adjusted *R* ^2^ = 0.069, *F*_4,121_; *p* = 0.068). The *group* consisting of three levels was not employed as covariate because it would have required additional dummy variables, potentially reducing the test's statistical power due to consumption of more degrees of freedom.

### 4.2 Interpersonal Relationships

**FIGURE 1:**
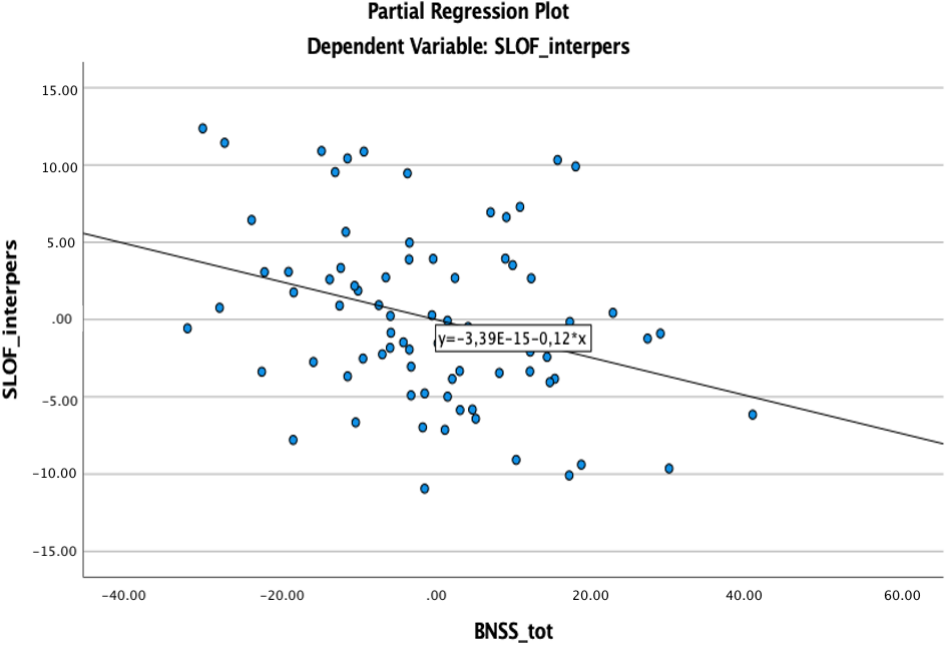
Partial regression plot showing a negative correlation between the SLoF *Interpersonal Relationships* subscale and the BNSS total score in the psychotic groups (DEL–SCZ + SCZ); BNSS_tot, the Brief Negative Symptoms Scale total score; SLOF_interpers, the Specific Levels of Functioning scale (SLoF) interpersonal functioning.

Considering the *Interpersonal Relationships* subscale, no significant differences were found between the SCZ and the DEL–SCZ groups, and the variances resulted to be equal (Levene's test not significant); thus, the simultaneous linear regression was implemented over all patients with psychosis, regardless of the underlying genetic condition (DEL–SCZ and SCZ; *N* = 82). This model, employing as predictors the *BNSS total score*, the *PANSS positive symptom*, the *PANSS general psychopathology* and the *MCCB composite score*, the *group* consisting of two levels as covariate, and the SLoF *Interpersonal Relationships* subscale as the dependent variable, resulted to be significant (adjusted *R* ^2^ = 0.291; *F*_5,76_ = 5.99, *p* < 0.001). As shown by beta standardized coefficients, the *BNSS total score* predictor had a significant impact on the *Interpersonal Relationships* variable (beta = −0.355; *p* = 0.003) (Figure 1). The beta coefficients of the other variables were not significant.

Another simultaneous linear regression analysis employing the SLoF *Interpersonal Relationships* subscale as dependent variable was implemented on the same psychotic group (*N* = 83). The model employing as predictors the *BNSS avolition*, the *BNSS distress*, the *BNSS alogia*, the *BNSS blunted affect*, the *BNSS apathy* and the *BNSS asociality* was significant (adjusted *R* ^2^ = 0.287; *F*_7,75_ = 5.087, *p* < 0.001); as shown by beta standardized coefficients, the *BNSS asociality* predictor had a significant impact on the *Interpersonal Relationships* variable (beta = −0.487; *p* = 0.038).

## 5 Discussion

The present study sought to investigate real‐life and social functioning in individuals with 22q11.2DS compared with a group of schizophrenic patients matched for age, sex and neurocognitive profile. We focused on social functioning, namely *Interpersonal Relationships* and *Social Acceptability*, considering that these dimensions would appear less influenced by environmental factors compared with other real‐life functioning domains. Thus, we investigated the impact of neurocognitive and psychopathological variables on patients' social functioning. To note, the matching procedure likely minimized the differences between groups for the match variables.

Regarding the socio‐demographic characterization of the sample, people with 22q11.2DS and patients with schizophrenia did not differ in their educational levels likely due to the structure of the national school system, which provides tailor‐cut assistance to individuals with clinical or intellectual disabilities. Both groups with psychosis (DEL–SCZ and SCZ) did not differ on psychopathological scores. Even if it appears counterintuitive, the sample matching procedure may partially explain this result because recruited individuals were homogeneous for age and cognitive profile. Moreover, DEL–SCZ and SCZ groups did not differ in antipsychotic treatment exposure, age of onset and illness duration suggesting that these groups were clinically comparable in their illness course (Table 5), although literature reports an earlier age of onset for people with 22q11.2DS (Vorstman et al. 2006).

**TABLE 5:** Demographical and clinical variables of the recruited sample (categorical variables).

*Categorical variables*	DEL–SCZ	SCZ	DEL	*χ* ^2^	*p*
	*N* = 19	*N* = 63	*N* = 44		
Sex, *N*. female (%)	3 (15.8%)	18 (28.6%)	15 (34.1%)	2.178	0.337
Treatment					
no ap	0.0% (*N* = 0)	6.3% (*N* = 4)		2.740	0.433
fga	10.5% (*N* = 2)	3.2% (*N* = 2)			
sga	84.2% (*N* = 16)	85.7% (*N* = 54)			
fga + sga	5.3% (*N* = 1)	4.8% (*N* = 3)			

Abbreviations: DEL, 22q11.2 deletion syndrome; DEL–SCZ, 22q11.2 deletion syndrome and schizophrenia; SCZ, schizophrenia; FGA, first‐generation antipsychotics; FGA + SGA, first‐ and second‐generation antipsychotics; NO AP, absence of antipsychotic treatment; *p*, statistical probability, significance; SCZ, schizophrenia; SGA, second‐generation antipsychotics; *χ* ^2^, Chi‐squared test.

Hence, we addressed our first aim to test the hypothesis of a similar real‐life functioning between a group of adults with 22q11.2DS and a matched group of patients with schizophrenia: DEL and SCZ groups did not differ in their everyday performances except for interpersonal functioning and work skills. Employing the *MCCB composite score* as a covariate, our results outlined differences and similarities between groups in their real‐life functioning: Although this variable significantly impacted patients' real‐life functioning, its influence has been controlled highlighting the effects of the independent variables. Interestingly, social functioning impairments in schizophrenia involve social cognition and social skills (Mueser et al. 1991). In particular, negative symptoms have been tightly associated with everyday‐life functioning impairment, more significantly than positive symptoms (Herbener and Harrow 2004). Similar results emerged from studies conducted in young people at risk of transition to psychosis, starting from schizotypal traits or prodromal and subthreshold symptoms (Lasalvia and Tansella 2012; Zoghbi et al. 2019).

The finding of similar functioning between a group of 22q11.2 individuals at risk of psychosis and a matched group of patients with schizophrenia led us to hypothesize the existence of factors influencing real‐life functioning independently of a full‐blown illness. Neurobiological, neurocognitive and neuropsychological factors, mainly related to genetic underpinnings and existing some time before the overt clinical onset, might be shared between 22q11.2DS and idiopathic schizophrenia, involving an overlap between clinical and functional features. These neurobiological factors seem to affect patients' real‐life functioning, as *Self Maintenance* and *Community Living Skills* domains, by means of their interactions with the environment. The evidence of higher working skills in DEL group compared with SCZ group might be partially explained by the existence of specific work placement programmes deserved to people with rare and compromising syndromes.

In the same line, patients with psychosis, regardless of their underlying genetic condition (DEL–SCZ and SCZ groups), did not differ on socio‐relational scores as assessed by the SLoF *Social Functioning* domain. Relational weakness seems to be tightly associated to psychotic disorders, both with a clear genetic aetiology and with multifactorial conditions, being related to patients' underlying neurocognitive profile. Finally, patients with 22q11.2DS without psychosis showed more preserved levels of real‐life functioning than patients with 22q11.2DS with psychosis but similar to patients with schizophrenia. We may suppose the existence of a synergistic effect between psychopathological symptoms and the microdeletion on both the *Self Maintenance* and *Community Living Skills* dimensions. Given that people with 22q11.2DS and psychosis and chronic schizophrenic patients displayed similar social functioning, a decline in the latter might be associated with an increased risk of psychotic onset in people with 22q11.2DS. Previous literature (Velthorst et al. 2017) corroborates this hypothesis, with young people with 22q11.2DS showing higher levels of introversion and isolation and greater internalizing behaviours leading to inability of social inclusion (Wagner et al. 2017) or greater schizotypal traits being associated with progressive social functioning impairment and psychosis (Fonseca‐Pedrero et al. 2016).

Regarding our second aim, we investigated the potential impact of psychopathology and neurocognition on the interpersonal functioning of patients with psychosis, regardless of their genetic condition (DEL–SCZ and SCZ groups). Negative symptoms, with particular regard to BNSS *asociality*, proved to be significant predictors of social functioning in patients with psychosis, confirming that a dimension of anhedonia/asociality/unwillingness influences patients' interpersonal abilities, likely arising from different pathophysiological mechanisms (Galderisi et al. 2014; Kimhy et al. 2006).

Our findings suggest that negative symptoms would impact patients' social functioning regardless of what is the genetic aetiology of their psychotic illness; this aspect may corroborate what has been previously reported about 22q11.2DS (Schneider et al. 2019), in line with evidence about both premorbid conditions and full‐blown psychoses (Herbener and Harrow 2004), particularly concerning apathy (Liemburg et al. 2013; Messinger et al. 2011; Ventura et al. 2013). Neurocognitive impairments and negative symptoms would predict real‐life functioning in schizophrenic patients, both through mediational or direct models (Davies and Greenwood 2020; Milev et al. 2005; Villalta‐Gil et al. 2006); a common aetiopathogenetic origin within negative symptoms, social cognitive deficits and interpersonal skills difficulties (Fett et al. 2011) might be hypothesized. Psychotic symptoms arising from an ascertained genetic basis on one side and a multifactorial condition on the other would share a common phenomenology similarly impacting patients' social functioning, thus shedding light on the neurobiological basis of schizophrenia through a better knowledge of its endophenotypes. Considering our results, we may hypothesize that a genetic condition like the 22q11.2DS, which is at higher risk of transition to psychosis compared with general population (Accinni et al. 2022; Bassett and Chow 2008; McDonald‐McGinn et al. 2015b), might involve premorbid factors which impact patients' real‐life functioning and coping abilities, well before the onset of a full‐blown psychosis. Such a consideration may apply also to patients with 22q11.2DS over 25 years of age, who therefore may be at a lower risk of transition; this syndrome would be thus confirmed as representing a biological opportunity to investigate neurocognitive and neuropsychological underpinnings that are endophenotypes associated with the vulnerability to psychosis, which in turn appears to be more multifactorial. These considerations have been further corroborated by looking at the social functioning of our sample, regardless of the underlying genetic condition. Psychotic disorders in a well‐defined genetic condition and chronic schizophrenia that instead is highly multifactorial showed overlapping impairments in patients' social functioning and interpersonal abilities, which appeared to be specific of a psychotic condition, as already reported (Galderisi et al. 2018).

Negative symptoms refer to impairments in affective tuning and emotional participation, likely preventing patients with psychosis from achieving a valid and adequate social life. Confirming this, negative symptoms, and in particular asociality, proved to constitute significant predictors of social functioning in the groups with psychosis, both with and without the 22q11.2 microdeletion. Therefore, social and interpersonal difficulties, as suggested by findings of a better social functioning in individuals with 22q11.2DS compared with patients with both deletion and psychosis, apparently would not result from the underlying genetic condition, although this remains only a speculation deserving further investigation.

Despite strengths, this study has several limitations; the main is the small sample size of 22q11DS groups, due to the rarity of the microdeletion. This has been addressed through adequate statistics and appropriate corrections. Another limitation was the application of sophisticated statistics only on the SLoF *Social Functioning* domain. The other SLoF domains, namely *Self Maintenance* and *Community Living Skills*, refer to patients' abilities particularly influenced by the environment and less determined by pure clinical conditions. We sought to investigate the impact of neuropsychological and neurocognitive variables on patients' social functioning which is tightly associated with clinical features. Moreover, the DEL group is heterogeneous, including patients with different psychiatric comorbidities or subthreshold psychotic symptoms, or at clinical high risk for psychosis, which were not assessed with specific instruments. One future objective will be recruiting more 22q11DS individuals to be able to separate another group of 22q11DS patients with attenuated psychotic symptoms for further studies. Another limitation of our study concerns the exclusion of the MCCB Attention/Vigilance domain from our analysis regarding individuals with 22q11.2DS; nevertheless, the MCCB total score, consisting of a composite score from different subscales and neurocognitive domains, which in turn are likely to be influenced by the attention/vigilance dimension, may have provided an implicit estimate of attention/vigilance abilities of 22q11.2 individuals. Finally, the present work consisted of a cross‐sectional observational study. A longitudinal design would better allow to describe more accurately the levels of real‐life functioning.

22q11.2DS looks like a reliable biological model to study the vulnerability to psychosis, referring both to clinical features and to their consequences on patients' real‐life and social functioning. Functional recovery of patients with psychosis should represent a primary aim of new therapeutic strategies and rehabilitation programmes, aiming at defining more effective intervention approaches that can improve several clinical outcomes (Lally et al. 2017; Roe, Mashiach‐Eizenberg and Lysaker 2011).

## Ethics Statement

The study adopted the Principles of Human Rights, as issued by the World Medical Association at the 18th WMA General Assembly, Helsinki, Finland, June 1964, and subsequently amended by the 64th WMA General Assembly, Fortaleza, Brazil, in October 2013 and received approval by the Ethics Committee of the of the Umberto I Hospital of Rome, protocol. 5341 n.250/19. All patient and other participant data were anonymized.

## Conflicts of Interest

The authors declare no conflicts of interest.

## Figures and Tables

**FIGURE A1:**
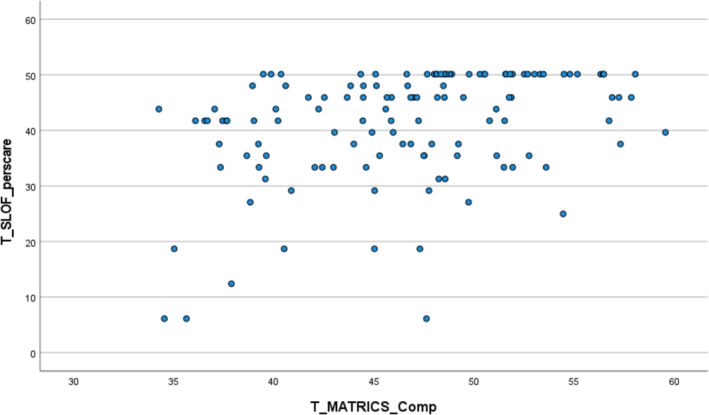
Scatterplots of the interaction between T_SLOF_perscare (SLoF Personal Care) and T_MATRICS_Comp (MCCB composite score).

**FIGURE A2:**
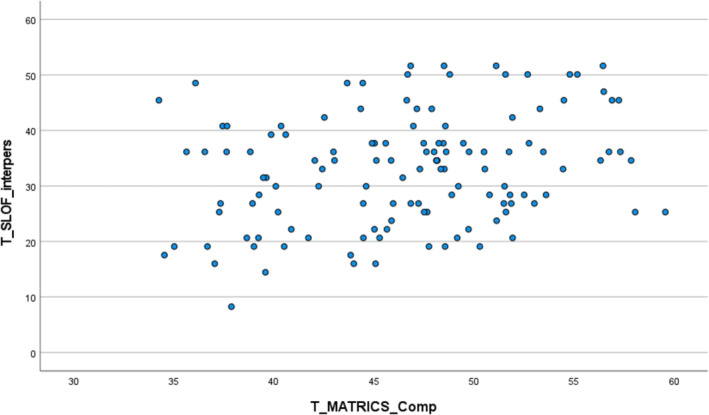
Scatterplots of the interaction between T_SLOF_interpers (SLoF Interpersonal Relationships) and T_MATRICS_Comp (MCCB composite score).

**FIGURE A3:**
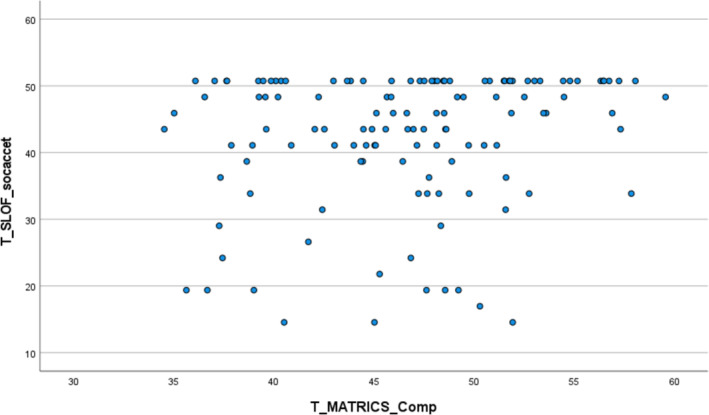
Scatterplots of the interaction between T_SLOF_socaccet (SLoF Social Acceptability) and T_MATRICS_Comp (MCCB composite score).

**FIGURE A4:**
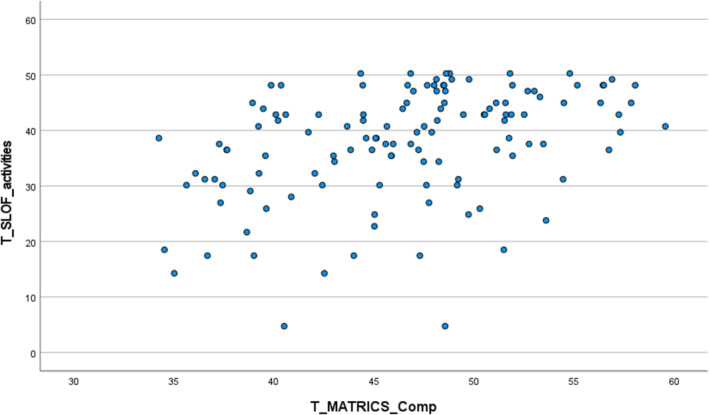
Scatterplots of the interaction between T_SLOF_activities (SLoF *Activities*) and T_MATRICS_Comp (*MCCB composite score*).

**FIGURE A5:**
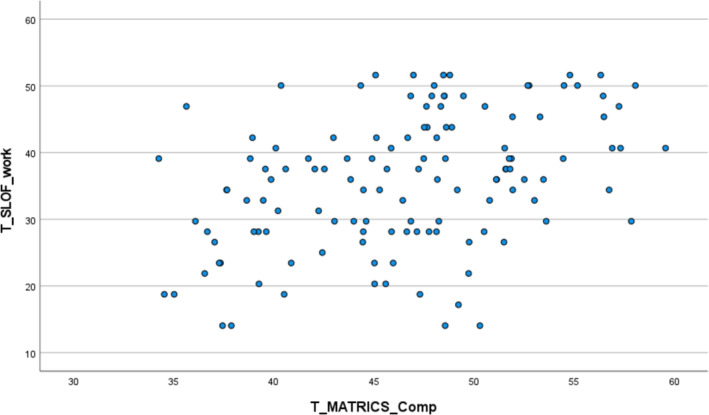
Scatterplots of the interaction between T_SLOF_work (SLoF *Work Skills*) and T_MATRICS_Comp (*MCCB composite score*).

## Data Availability

Aggregated data may be available from the corresponding author upon reasonable request. The data are not publicly available due to privacy or ethical restrictions.

## Appendix A

### A.1 MANCOVA Assumptions

Several assumptions have been tested before proceeding with the MANCOVA in order to ensure the validity of the analysis. Skewness and kurtosis indices ranged between 1 and −1 (Table A1) indicating that non‐normal distributions would not represent a significant bias (Muthén and Kaplan 1985; West, Finch, and Curran 1995).

**TABLE A1:** Skewness and kurtosis indices are presented.

	*N*	Skewness		Kurtosis	
	Statistic	Statistics	SE	Statistic	SE
*Pers. care*	126	−1.031	0.216	1.042	0.428
*Interpers*.	126	0.060	0.216	−0.705	0.428
*Social accept*.	125	−1.151	0.217	0.949	0.430
*Activities*	125	−1.072	0.217	0.928	0.430
*Work skills*	126	−0.229	0.216	−0.728	0.428
*MCCB comp*.	126	−0.063	0.216	−0.701	0.428
*Age*	126	1.024	0.216	1.053	0.428

Abbreviations: Pers. care, SLoF Personal Care Skills; Interpers., SLoF Interpersonal Relationships; Social accept., SLoF Social Acceptability; Activities, SLoF Activities; Work skills, SLoF Work Skills; MCCB comp, MCCB composite score; SE, standard error.

Levene's test for homoscedasticity of variances was performed: only the Activities subscale was significant (Table 3 in the main paper).

The interactions between the covariates *MCCB composite score* and *Age* and the independent variable *Group* were tested to ensure that the regression slopes were homogeneous: The interactions involving the dependent variables *Interpersonal Relationships* and *Social Acceptability* did not result significant; on the other hand, the interactions between the covariate *Age* and the variables *Personal Care* and *Work Skills* were significant, respectively, for the first and the second dummy term. Concerning the variable *Activities*, the interaction between the *MCCB composite score* and the first dummy term was significant (Table A2). These significant interactions were not deemed to be critical for the primary research outcomes, and thus, the assumption of homogeneity of regression slopes has been considered adequately satisfied for main analyses purposes.

**TABLE A2:** Assumptions of homogeneity of regression slopes are presented.

Subscale SLoF	*t*_(120)_ interaction 1 MCCB	Sig. interaction 1 MCCB	*t*_(120)_ interaction 2 MCCB	Sig. interaction 2 MCCB	*t*_(120)_ interaction 1 Age	Sig. interaction 1 age	*t*_(120)_ interaction 2 age	Sig. interaction 2 age
Pers. care	0.73	0.467	−1.29	0.200	2.41	0.017^a^	0.68	0.133
Interpers.	−0.15	0.878	−0.64	0.521	1.02	0.310	1.58	0.116
Social accept.	0.99	0.326	−1.17	0.244	0.08	0.938	0.69	0.493
Activities	−0.95	0.342	−2.42	0.017*	1.37	0.174	1.62	0.108
Work skills	0.51	0.607	0.69	0.490	1.72	0.089	3.52	0.001*

*Note:* The table shows *t* (degrees of freedom) and Sig. (*p*, significance) for each interaction; interaction 1 MCCB, *t* and *p* of Dummy1 × MCCB comp. interaction; interaction 2 MCCB: *t* and *p* of Dummy2 × MCCB comp. interaction; interaction 1 age: *t* and p of Dummy1 × age interaction; interaction 2 age: *t* and *p* of Dummy2 × age interaction.

Abbreviations: df, degrees of freedom; MCCB comp, MCCB composite score; SE, standard error; SLoF, Specific Level of Functioning Scale; Pers. care, SLoF Personal Care Skills; Interpers., SLoF Interpersonal Relationships; Social accept., SLoF Social Acceptability; Activities, SLoF Activities; Work skills, SLoF Work Skills.

^a^ Significant interaction terms.

Scatterplots of the interactions between the dependent variables and the covariates (Figures A1, A2, A3, A4, A5) resulted to show linear relationships, supporting the assumption of linearity.

Values of variance inflation factor (VIF) were calculated for the covariates and the independent variables to check for the multicollinearity: All VIF values resulted subthreshold (< 10), indicating no multicollinearity issues.

**Table:** TABLE A3. Multicollinearity statistics; dependent variable: SLoF *Personal Care*.

	Tolerance	VIF
Group	0.951	1.052
Age	0.969	1.032
MCCB comp.	0.951	1.052

Abbreviations: MCCB comp, MCCB composite score; SLoF, Specific Level of Functioning Scale.

**Table:** TABLE A4. Multicollinearity statistics; SLoF Interpersonal Relationships.

	Tolerance	VIF
Group	0.951	1.052
Age	0.969	1.032
MCCB comp.	0.951	1.052

Abbreviations: MCCB comp, MCCB composite score; SLoF, Specific Level of Functioning Scale.

**Table:** TABLE A5. Multicollinearity statistics; SLoF Social Acceptability.

	Tolerance	VIF
Group	0.951	1.051
Age	0.969	1.033
MCCB Comp.	0.951	1.052

Abbreviations: MCCB comp, MCCB composite score; SLoF, Specific Level of Functioning Scale.

**Table:** TABLE A6. Multicollinearity statistics; dependent variable: SLoF Activites.

	Tolerance	VIF
Group	0.951	1.051
Age	0.967	1.034
MCCB Comp.	0.950	1.053

Abbreviations: MCCB comp, MCCB composite score; SLoF, Specific Level of Functioning Scale.

**Table:** TABLE A7. Multicollinearity statistics; dependent variable: Work Skills.

	Tolerance	VIF
Group	0.951	1.052
Age	0.969	1.032
MCCB comp.	0.951	1.052

Abbreviations: MCCB comp, MCCB composite score; SLoF, The Specific Level of Functioning Scale.

### A.2 MANCOVA Multivariate Tests

**TABLE A8:** Complete MANCOVA multivariate tests with effects of fixed factors, group variables and covariates *Age* and *MCCB composite score*.

	Value	*F*	Hypothesis *df*	Error *df*	Sig.	Partial *η* ^2^
Test statistics for *Group*						
Wilk's lambda	0.560	7.73	10	231	< 0.001	0.251
Pillai's trace	0.502	7.77	10	229	< 0.001	0.251
Hotelling's trace	0.674	7.69	10	227	< 0.001	0.252
Roy's largest root	0.393	9.11	5	114	< 0.001	0.282
Test Statistics for *Age*						
Wilk's lambda	0.936	1.57	5	114	0.174	0.064
Pillai's trace	0.064	1.57	5	114	0. 174	0. 064
Hotelling's trace	0.068	1.57	5	114	0. 174	0. 064
Roy's largest root	0.068	1.57	5	114	0. 174	0. 064
Test statistics for *MCCB composite score*						
Wilk's lambda	0.804	5.6	5	114	< 0.001	0.196
Pillai's trace	0.196	5.6	5	114	< 0.001	0.196
Hotelling's trace	0.243	5.6	5	114	< 0.001	0.196
Roy's largest root	0.243	5.6	5	114	< 0.001	0.196

Abbreviation: *df*, degrees of freedom.
